# Superhydrophobic silica wool—a facile route to separating oil and hydrophobic solvents from water

**DOI:** 10.1088/1468-6996/15/6/065003

**Published:** 2014-11-12

**Authors:** Colin R Crick, Davinder S Bhachu, Ivan P Parkin

**Affiliations:** Materials Chemistry Research Centre, Department of Chemistry, University College London, 20 Gordon Street, London WC1H OAJ, UK

**Keywords:** superhydrophobic, oil spill, water, separation, oleophilic

## Abstract

Silica microfiber wool was systematically functionalized in order to provide an extremely water repellent and oleophilic material. This was carried out using a two-step functionalization that was shown to be a highly effective method for generating an intense water repulsion and attraction for oil. A demonstration of the silica wools application is shown through the highly efficient separation of oils and hydrophobic solvents from water. Water is confined to the extremities of the material, while oil is absorbed into the voids within the wool. The effect of surface functionalization is monitored though observing the interaction of the material with both oils and water, in addition to scanning electron microscope images, x-ray photoelectron spectroscopy and energy dispersive x-ray analysis. The material can be readily utilized in many applications, including the cleaning of oil spills and filtering during industrial processes, as well as further water purification tasks—while not suffering the losses of efficiency observed in current leading polymeric materials.

## 1. Introduction

Recent reports on oceanic oil spills have highlighted the devastating effect that these events can have on the environment and their subsequent economic consequences [1]. The dependence that human populations have for oil ensures that its extraction will be a result of deeper drilling, and other forms of hazardous abstraction methods. This heightened risk not only renders large oil spills more likely, but the locations of these accidents will be ever more remote [2]. This almost inevitable threat to the environment and the finances of nations and individuals has driven the development of technology to tackle this problem. There are many common approaches currently used to separate oil–water mixtures [3–5], however the selective filtration of water–oil mixtures can be successfully achieved when a material has simultaneous repulsion of water and attraction of oil (or vice versa) [5]. When contemplating the removal of oil from water, important in oil spills into sea water and separation during processing, then a material best suited to filtration is one that greatly repels water (superhydrophobic) but greatly attracts oils (superoleophilic). This ensures that oil is captured within the material while the water is not [6].

Surface hydrophobicity is maximized through combining a material that has inherently water repelling properties, in conjunction with an extremely high surface roughness. Water repelling species, such as alkyl or fluorinated alkyl groups, act to lower surface energy and will repel water molecules [7]. The surface roughness acts to magnify these properties, while allowing air to be trapped under surface protrusions; both features act to increase the hydrophobicity of the surface [8]. Alkylated surface groups also act to strongly attraction oils [9]. Porous materials that utilize this hydrophobic/oleophilic property would be able to repel water from the bulk of the material while attracting in oil.

The main measure of surface hydrophobicity is the water contact angle. This is the angle made between the plain of a surface and tangent made by a water droplet laying on the surface at the water-surface–air interface. A surface is termed superhydrophilic if this angle is below 5°, hydrophilic if it is below 90°, hydrophobic if it is greater than 90° and superhydrophobic above 150° [10]. Models for the visualization of surface hydrophobicity have been reported; the Cassie–Baxter model rationalizes the trapping of air under water lying on a surface (this is the case in most superhydrophobic surfaces) [8]. Superhydrophobicity can also be gauged through water bouncing experiments [11]. The same principle of surface design can apply for hydrophobic solvents, where oleophilic surfaces give contact angles below 90°; additional roughness will magnify the attraction of oils to the surface [12]. In order to facilitate water–oil separation a surface must have both low contact angles for oils or hydrophobic solvents, and high contact angles with water. Another requirement is that the surface should be highly porous to facilitate the absorption of solvent into the bulk where it can be retained.

The design, manufacture and application of superhydrophobic surfaces are all well reported in the literature [13–15]. There are also device components and commercial products currently on the market, which include foams, meshes/filters and dispersants [16–18]. Most of these technologies are based on superhydrophobic/superoleophilic characteristics or vice versa. General routes to making superhydrophobic surfaces include the roughening of already water repellent surfaces, coating/functionalization of already roughened surfaces, in addition to the development of surface roughness using already water repelling material [13–15]. Separating oil–water mixtures requires further developments on the above approaches. The superhydrophobic surfaces will repel water, but must also attract oil, thus there must be somewhere for that oil to go; either into the bulk of the material, or an alternative pathway must be provided [19, 20]. The literature reports the use of superhydrophobic meshes which allow oils to drip through the pores, leaving water on the top side of the mesh and the oil to drip into a collector [5]. Another reported approach is the use of polymeric materials which preferentially absorb oil over water, however the hydrophobic solvents used could not be easily separated from the polymer which was disposed of after use [21]. This highlights a major failing of some approaches to separating oil from water; removal of the oil from the material after separation has occurred. Recent reports also include the fabrication of water purification devices [22–24]. These are enclosed systems which use a flow of solvent/water mixtures through a superhydrophobic or superoleophobic filter. This method of purification has been shown to be highly effective, however these systems are susceptible to blockages if the denser liquid is not absorbed by the filter. For example using a water/hexane mixture on a hydrophobic filter—the denser water would sink to cover the filter thus not allowing the collection of hexane. These systems would not be readily applicable to large oil spills (relative to uncomplicated oil absorbent materials), as a pumping mechanism or some other alternative would be required. Superhydrophobic/oleophilic sponges have also been reported, these are materials that can absorb oil while repelling water [25–27]. These can show extremely selective absorption with respect to oils and hydrophobic solvents, however the materials used in their fabrication can suffer from degradation and polymer swelling, in addition to high fabrication cost [28–30].

**Figure 1.**
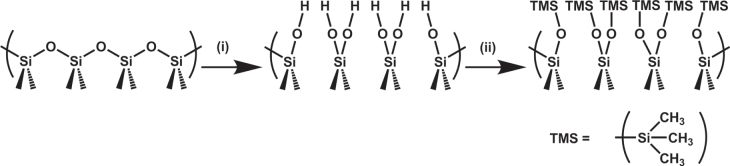
Surface reaction scheme at silica surface. Surface silanol groups are formed under the action of piranha solution (i), and subsequently functionalized by using HMDS to form surface TMS groups (ii). The surface undergoes a hydrophilic to hydrophobic transformation during *reaction ii*.

We report the functionalization of silica (SiO_2_) wool. The surface of the silica wool was activated with piranha solution and then functionalized using hexamethyldisilazane (HMDS), to form trimethylsiloxane (TMS) groups on the surface of the wool (figure 1) [31]. The result was a lowered surface energy, which when combined with the inherent surface roughness of the wool, gave a superhydrophobic material. The surface functionalization also rendered the material superoleophilic, strongly attracting hydrophobic solvents and oils (toluene, hexane, petroleum ether and motor oil). The result was a material that could separate oil and hydrophobic solvents from water with high efficiency. The range of solvents used could be easily removed from the wool by compression after use, enabling the wool to be reused. This study shows the relative absorption efficiencies of each solvent and also the separation experiments with water–solvent mixtures.

## 2. Experimental

### 2.1. Characterization techniques

Scanning electron microscopy (SEM) images were recorded on a Jeol JSM-6301F microscope operating at 5 kV. Samples were vacuum sputtered with a very thin film of gold to improve surface electrical conductivity. Energy dispersive x-ray analysis was carried out on the same machine, using an accelerating voltage of 15 kV and uncoated samples. X-ray photoelectron spectroscopy (XPS) measurements were performed with a Thermo Scientific K-alpha system using monochromatic Al-K_*α*_ radiation. Survey scans were collected in the range 0–1100 eV (binding energy) at a pass energy of 160 eV. Higher resolution scans were recorded for the principal peaks of Ti (2p), Nb (3d), O (1s), C (1s) and Si (2p) at a pass energy of 20 eV. Peak positions were calibrated to carbon and plotted using the CasaXPS software. IR spectroscopy was also employed using a Perkin Elmer FT-IR (Fourier transform infrared) Spectrum RX1 instrument.

Water contact angle measurements were carried out using an FTA-1000 drop shape instrument; 3 *μ*L water droplets were used and the contact angle of the water droplet was directly observed.

### 2.2. Materials

Motor oil (*Brand*—Formula 1, 20w/50) was purchased from Fisher Scientific. All other chemicals and substrate materials used in this investigation were purchased from Sigma-Aldrich Chemical, including glass wool (for laboratory use), concentrated sulfuric acid (95.0–98.0%), hydrogen peroxide (35% solution in water) and HMDS (≥99%). Laboratory solvents were purchased from Fisher Scientific and of the highest possible grade.

### 2.3. Silica wool functionalization

The functionalization of the wool was carried out via two steps. Firstly wool portions (of various masses) were stirred in piranha solution (3:1 concentrated sulfuric acid to 35% hydrogen peroxide solution) for 5 min, the portions were rinsed thoroughly with deionized water to remove any piranha solution, and then acetone to remove water and facilitate drying. The wool portions were then placed in a drying oven at 80 °C for 30 min. The dried portions were then stirred for 24 h at 40 °C in a solution of HMDS in toluene (10% v/v). This treatment acts to change the surface silanol groups of the silica to trimethylsiloxane groups. The functionalized portions of wool were thoroughly washed with toluene and then air dried overnight. The dried silica wool was then used in hydrophobic solvent absorption experiments.

### 2.4. Oil–water separation testing

Superhydrophobic wool was submerged in toluene, hexane and petroleum ether. Later experiments used motor oil in similar experiments. The soaked wool portions were weighed, and amount of solvent absorption per unit weight of wool was assessed. The reusability of the wool samples was assessed by compressing wool samples between two glass surfaces (with manual compression, schematic shown in supplementary information S1, available at stacks.iop.org/STAM/15/065003/mmedia), removing as much solvent as possible, and repeating this process.

Mixtures of water and toluene or motor oil (hydrophobic fluids) were then separated using further portions of wool. These hydrophobic fluids, which floated atop the water, were selectively absorbed into the voids of the mesh. The mixture, containing the silica wool sample, was stirred briefly and removed using tongs. The soaked wool was then immediately put into a separate container. The volume of remaining water and hydrophobic fluid was quantified by the volume remaining in the initial flask.

### 2.5. Water bouncing measurement

The dynamic interaction of water with wool samples was analysed through water bouncing experiments. A syringe equipped with a dispensing tip was used to give generate 8 *μ*L water droplets. These droplets were dropped from a height of 20 mm onto the wool. This dynamic measurement provides an alternative indication of surface hydrophobicity [11]; highly significant to the wool samples as the undulant nature of the surface reduced the ability to observe the static water contact angle, by disrupting the line-of-sight to the air–water-surface interface.

## 3. Results and discussion

**Figure 2.**
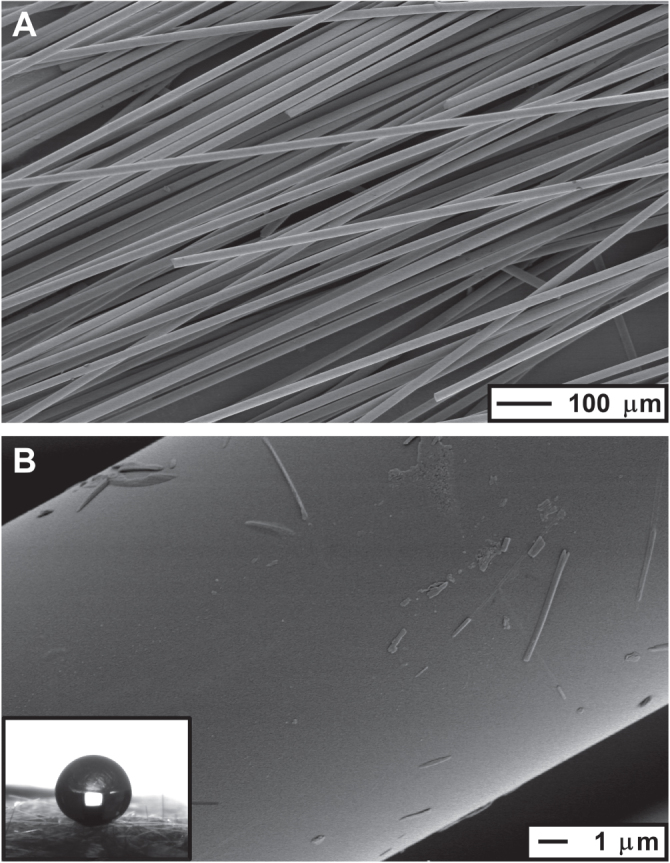
SEM images of functionalized silica wool. The fibres measure ∼16 *μ*m in diameter. The individual fibres have a relatively smooth surface (B), and so roughness comes from the collection of fibres within the wool (A). The inset shows a water droplet (3 *μ*L) on silica wool functionalized using HMDS. Water contact angles were above 165°.

Preliminary experiments were carried out using small portions of wool (∼0.025 g); these samples were imaged *via* SEM before and after any treatments were applied. There was no observed change in the physical appearance or robustness of the silica wool, and no change in microstructure (figure 2); this suggested that any change to the material was surface chemistry focused. The SEM images did show the inherent roughness of the wool, with silica fibres approximately 16 *μ*m in diameter. The silica wool was calculated to have a surface area of approximately 0.1 m^2^ g^−1^.

The as-received silica wool was hydrophilic; any water droplets making contact with the surface were immediately absorbed into the voids of the material. This was not altered by the action of piranha solution, as water absorption happened as readily; rendering water contact angle measurements inapt for describing the hydrophobicity of the material. The wool was rendered superhydrophobic upon functionalization *via* the overnight treatment with HMDS (figure 1, embedded). Water contact angles of approximately 165° were observed, however exact determination was made difficult as the surface of the wool was uneven. Water droplets would tend to roll off the surface of the wool when tilted away from the horizontal (see supplementary information S2, available at stacks.iop.org/STAM/15/065003/mmedia), however trapping of water droplets was common where wool strands intersected. In order to gain a further evaluation of the superhydrophobicity of the wool water bouncing experiments were carried out. The observed total number of water bounces averaged 3–4 (using standard conditions) [11], which correlates with the estimated water contact angle (∼165°).

**Figure 3.**
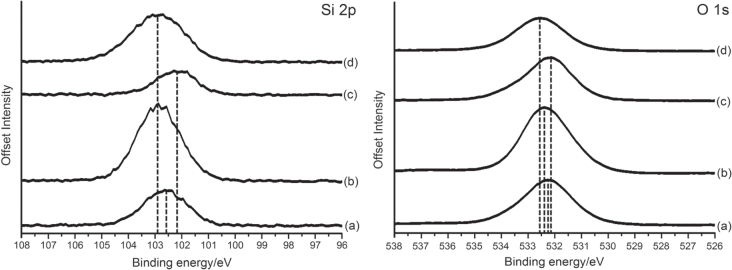
Si (2p) and O (1s) XPS spectra of (a) as-bought silica wool, (b) HMDS functionalized as-bought silica wool, (c) piranha pre-treated silica wool and (d) HMDS functionalized piranha pre-treated silica wool. Dotted lines show the centre of each signal.

Figure 3 shows the x-ray photoemission spectra for Si (2p) and O (1s) core transitions for untreated/treated silica wool. The Si (2p) spectra are presented in figure 3 (on the left) and show that for untreated and piranha treated silica wool that surface SiOH groups are present in significant quantities. Silica wool pre-treated with piranha solution (figure 3(c)) shows a shift to lower binding energy that correlates to a more electropositive environment around the Si. It can be seen that the O 1s position is also shifted towards lower binding energy (figure 3(c)) with piranha treatment indicating a change in the surface oxygen environment. A broad shoulder can also be seen at higher binding energy, indicative of surface hydroxyl species. This feature is more pronounced in the case of silica wool pre-treated with piranha solution. The experiments were repeated in order to determine experimental reproducibility, as the observed shifts are not very large. Infra-red spectroscopy was carried out before and after functionalization, however no change in surface was able to be detected. This was a limitation of the infra-red instrument used.

The absorption of different hydrophobic solvents was tested (table 1). Portions of wool of recorded mass were submerged into excess amounts of water, toluene, hexane or petroleum ether, the samples were withdrawn and weighed immediately. It was found that in all cases water absorption into the wool was significantly lower than that of the three hydrophobic solvents used. The efficiency of wool samples to absorb solvents increased with the mass of the wool sample used. The relative amount of water picked up by the wool also greatly decreased with increased mass. The reason for this is that the little water that was collected was mainly trapped at the surface of the wool, as it did not enter the bulk. Furthermore, with increasing size the surface area-volume ratio decreases, thus there is a relatively greater volume to absorb solvent and less surface to trap water. The three hydrophobic solvents all show similar patterns in absorption volume. The best performing solvent was hexane which was absorbed into the wool over 38 times that of water, when using 10 g of superhydrophobic wool. The ability of the oleophilic wool to absorb and retain each solvent is dependent on three main factors. Firstly, the surface attraction for the respective solvent: the stronger the attraction the more solvent will be absorbed and will be held with a stronger force. Secondly, the viscosity of each solvent affects the amount of solvent collected. This is due to the seeping of solvent into the solvent mixture upon the removal of wool samples. Solvents with lower viscosity would demonstrate a faster flow from the soaked silica wool samples. A final factor that influences the volume of solvent collected is the room temperature density of the respective solvents (Toluene 0.87 g cm^−3^, hexane 0.68 g cm^−3^ and petroleum ether 0.64 g cm^−3^). The density of solvents would affect the absorption efficiency, as the voids in the silica wool samples would hold a greater mass of a denser solvent.

**Table 1.** Table showing the absorption of hydrophobic solvents and water into functionalized portions of wool. Measurements were repeated three times for each wool portion.

	0.025	1.5	10
Mass of wool (g)	Average volume of solvent absorbed per gram of silica wool (mL)		
Water	3.33	1.37	0.32
Toluene	7.73	10.02	11.61
Hexane	8.08	11.53	12.33
Petroleum ether	5.54	10.03	10.66

The ‘as-received’ and hydroxylated wool samples did not have contrasting interactions with oil and water, as shown for superhydrophobic samples. Equivalent experiments carried out using 0.025 g potions of ‘as-received’ wool showed that it was able to absorb 8.4 mL g^−1^ of toluene and 11.5 mL g^−1^ of water; whereas hydroxylated wool absorbed 3.9 mL g^−1^ of toluene and 5.9 mL g^−1^ of water. Silica wool samples exposed to only the HMDS functionalization showed absorption of 7.6 mL g^−1^ and 6.5 mL g^−1^ of toluene and water respectively. The ‘as-received’ and hydroxylated samples absorbed more water than toluene, rendering those samples inadequate for the purpose of scavenging oil from water. The wool sample which solely underwent HMDS treatment, had similar affinities for both toluene and water. The latter experiment showed the importance of the initial hydroxylation; this is proposed to produce a greater density of surface silanol groups [32], which then in turn produce a higher density of TMS groups on the surface. The higher density of TMS groups results in a more hydrophobic material, and thus a material with greater selective absorption of oils [33].

**Figure 4.**
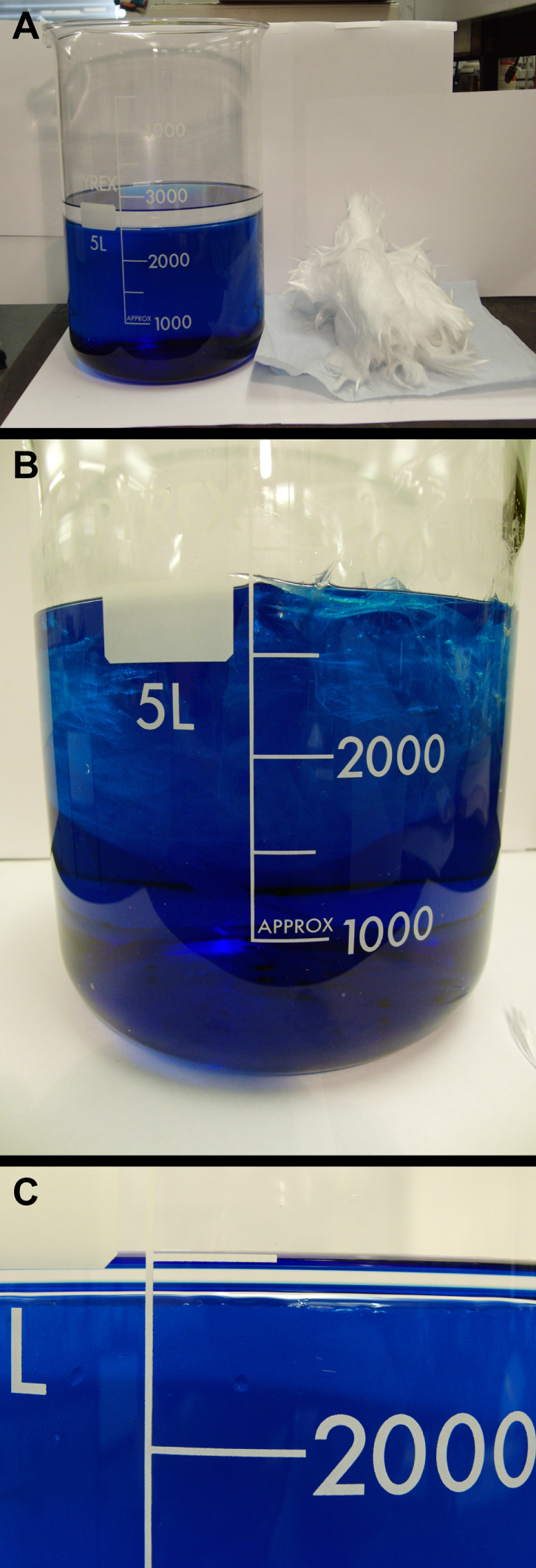
A series of photographs showing the removal of toluene from water. (a) A 50 g portion of superhydrophobic silica wool was used; the starting solvent mixture contained 2500 mL of water and 250 mL of toluene. (b) The wool was submerged into the mixture absorbing the toluene (no organic layer present). (c) The wool was removed; the end mixture contained ∼2450 mL of water and 40 mL of toluene. The water was coloured with low concentrations of methylene blue dye to aid visualization (this did not alter the results obtained).

The ability of the wool to separate out hydrophobic solvents from water was also tested. A 50 g portion of wool was used to separate 250 mL of hydrophobic solvent from 2500 mL of water (figure 4). The superhydrophobic wool samples were completely submerged in the solvent mixture, withdrawn, and remaining solvent measured. The results were similar to those seen in the individual testing, with very low amounts of water trapped on the wool’s surface (average ∼1 mL g^−1^). The amounts of toluene, hexane and petroleum ether absorbed into the 50 g mesh were 210 mL, 213 mL and 204 mL respectively. The superhydrophobic wool samples did not pick up 100% of the solvent as there was some amount of dripping back into the water-solvent container.

Portions of superhydrophobic wool (∼10 g) were used to assess reusability of the wool. The same wool sample was used repeatedly to pick up hydrophobic solvent, removed and compressed by hand between two glass plates. The mass of solvent removed with each of ten sequential submersions was recorded. Each experiment was repeated three times. The results showed that the first submersion of the wool picked up the most solvent, 12.5 mL g^−1^ was averaged for hexane. This steadily decreased to 7.6, 7.2 and 6.2 mL g^−1^ in the subsequent submersions, until after five submersions when an average of 5.4 mL g^−1^ was removed each time. Similar results were observed with the other solvents used, with 5.7 mL g^−1^ reached for toluene and 4.8 mL g^−1^ for petroleum ether. This shows the hydrophobic silica wool can be used repeatedly to remove hydrophobic solvents.

The wool samples were examined through water contact angle, SEM and EDX analysis after the absorption experiments. No change in microstructure or composition was observed after separation experiments were carried out. Water contact angles remained unchanged and there was no visual modification of the material observed. Surface hydrophobicity measurements (water contact angle and bouncing) were taken after samples were cleaned, and three months after use; no degradation of hydrophobicity was observed.

The wool samples were also exposed to thicker motor oil (20 w/50). Similar results were observed; the wool was able to absorb >10 mL of oil per gram of superhydrophobic wool. The wool was also able to be reused in collecting the motor oil: the average collection of a 10 g sample of wool after five consecutive uses was 6.9 mL g^−1^. The wool was also tested to separate the motor oil from water (see supplementary information S3, available at stacks.iop.org/STAM/15/065003/mmedia). The collection proved slightly more efficient as there was substantially less dripping from the wool when pulled from the mixture. The higher efficiency was caused by the reduced tendency of the thicker motor oil to drip (with respect to the faster flowing hydrophobic solvents) once captured in the wool and removed from the mixture.

The separation of oil from water using the reported superhydrophobic silica wool operates *via* preferential absorption of oil into the voids of the wool material. This can be compared to materials such as activated carbon, where organic impurities in water can be absorbed into the voids within the microstructure [34]. A substantial benefit that superhydrophobic silica wool has over this and related materials is that the removal of oil can be performed by simple and repeatable compression; without the need for rinsing. This extends the potential areas of application beyond static filtration materials and toward usage as boom materials for oil absorption at sea, due to the ease of reusability. Currently in this area; polyurethane foams are amongst the most successful and widely used [35], however current limitations of these materials are reflected in the swelling of the polymer on extended exposure to solvents and oils [29]. The result of this is that the absorbed oil is trapped between polymer chains and will not be able to be easily recovered. In addition, the ability of polyurethane to absorb oil will be diminished over time; a factor that does not affect superhydrophobic silica wool. The effectiveness of these materials could also be improved through the incorporation of additional porosity or roughness [36, 37].

## 4. Conclusions

The material reported in this article demonstrates highly efficient and robust oil–water separation. The superhydrophobic silica wool was capable of achieving water contact angles of 165° on average. The wool, which is readily produced using affordable methods, strongly absorbs hydrophobic solvents and thick oils (up to 12.5 mL g^–1^ of wool). The ability to reuse the material was effectively demonstrated, acting as a sponge, able to absorbed the oils and repel water. The wool samples could be rapidly incorporated in a commercial device, either in the use of cleaning oil spills or essentially in other areas where hydrophobic solvents and oils would need to be separated from water.
